# The Role of E-Cadherin in Maintaining the Barrier Function of Corneal Epithelium after Treatment with Cultured Autologous Oral Mucosa Epithelial Cell Sheet Grafts for Limbal Stem Deficiency

**DOI:** 10.1155/2016/4805986

**Published:** 2016-09-29

**Authors:** Fawzia Bardag-Gorce, Richard H. Hoft, Andrew Wood, Joan Oliva, Hope Niihara, Andrew Makalinao, Jacquelyn Thropay, Derek Pan, Imara Meepe, Kumar Tiger, Julio Garcia, Amanda Laporte, Samuel W. French, Yutaka Niihara

**Affiliations:** Los Angeles Biomedical Research Institute (LA BioMed), Harbor UCLA Medical Center, Torrance, CA 90502, USA

## Abstract

The role of E-cadherin in epithelial barrier function of cultured autologous oral mucosa epithelial cell sheet (CAOMECS) grafts was examined. CAOMECS were cultured on a temperature-responsive surface and grafted onto rabbit corneas with Limbal Stem Cell Deficiency (LSCD). E-cadherin levels were significantly higher in CAOMECS compared to normal and LSCD epithelium. Beta-catenin colocalized with E-cadherin in CAOMECS cell membranes while phosphorylated beta-catenin was significantly increased. ZO-1, occludin, and Cnx43 were also strongly expressed in CAOMECS. E-cadherin and beta-catenin localization at the cell membrane was reduced in LSCD corneas, while CAOMECS-grafted corneas showed a restoration of E-cadherin and beta-catenin expression. LSCD corneas did not show continuous staining for ZO-1 or for Cnx43, while CAOMECS-grafted corneas showed a positive expression of ZO-1 and Cnx43. Cascade Blue® hydrazide did not pass through CAOMECS. Because E-cadherin interactions are calcium-dependent, EGTA was used to chelate calcium and disrupt cell adhesion. EGTA-treated CAOMECS completely detached from cell culture surface, and E-cadherin levels were significantly decreased. In conclusion, E cadherin high expression contributed to CAOMECS tight and gap junction protein recruitment at the cell membrane, thus promoting cellular adhesion and a functional barrier to protect the ocular surface.

## 1. Introduction

Ocular surface regeneration with cultured oral mucosa epithelial cells has been used for almost a decade. The efficacy of this technique has been successfully demonstrated by investigators who have used various methods for preparing and delivering the graft to the ocular surface [1–6]. Investigators have also studied the epithelial barrier function of cultured oral mucosal or limbal epithelial cell sheets [4, 6, 7]. However, these reports are still vague about the definition of an epithelial barrier function. Does a barrier function include a barrier to conjunctivalization of the cornea, a barrier to external toxic and infectious agents, and/or barriers to neo-vascularization? These are questions that have not been answered. The reports that have investigated oral mucosal epithelial cell sheets barrier function documented various results. Satake et al. 2008 [4] and Shimazaki et al. 2009 [7] cultured oral mucosa epithelial cells (OMECS) on amniotic membrane and reported that OMECS tissue was permeable to fluorescein and that limbal stem cells had a better barrier function than cultured oral mucosal epithelial cells, respectively. However, Hori et al. 2008 [8] and Hayashi et al. 2010 [9] cultured OMECS on temperature-responsive surfaces (CellSeed Inc., Tokyo, Japan) and, using MUC16 and ZO-1 staining, reported a functional barrier. Recently, Duncan et al. 2015 [10] used Corning inserts (Corning Inc., NY) to culture OMECS and used Quantum Dot to report a functional barrier. In the present study, we used assays for E-cadherin and other proteins associated with intercellular adhesion to examine the epithelial barrier of CAOMECS grafts. We investigated adhesion proteins and junctional complexes before and after grafting onto corneas with LSCD. The presence of normal cell-to-cell junctional complexes is critical to the efficacy and safety of CAOMECS. Cell-to-cell adhesion includes adherens, tight and gap junctions (AD, TJ, and GJ, resp.), all of which contribute to maintaining epithelial integrity. Cadherin proteins are adhesion proteins that control cell contacts and cell motility [11]. While E-cadherin extracellular domains mediate Ca^2+^-dependent cell-cell binding, their intracellular domains recruit beta-catenin proteins, which in turn interact with actin cytoskeleton filaments, promoting adhesiveness [12], thereby limiting destabilization of cell junctional complexes, and contributing to the epithelial barrier function.

The temperature-sensitive culture plate developed by CellSeed, Inc., (Tokyo, Japan) allows CAOMECS harvesting in a tissue-like multilayered sheet ready for grafting onto the recipient cornea [1, 13]. The harvested cell sheets contain intact extracellular matrix (ECM) that increase adhesions between CAOMECS graft and the recipient corneal surface. Our previous study using microarray analysis of CAOMECS gene expression [14] demonstrated that gap junction genes Connexin 43 (Cnx43) and Connexin 45 were upregulated. The upregulation of these two gap junction proteins suggested that cell-to-cell interactions were at least partially functional. We also examined E-cadherin signaling, as it is essential for cytoskeleton organization, cell adhesion, and functions as a suppressor of cell proliferation/migration [15, 16]. The expression of membranous E-cadherin levels favors the formation of TJ and GJ complexes by recruiting beta-catenin, which in turn recruit alpha-catenin, and cytoskeleton filaments that interact with ZO-1 and finally with Cnx43. This E-cadherin signaling increases cell-to-cell interactions to promote cell adhesion and probably improve the epithelial barrier function. Part of beta-catenin pool is phosphorylated in the cytoplasm and then degraded by the proteasome pathway [17]. If beta-catenin is not phosphorylated, it translocates to the nucleus and stimulates Wnt pathway gene expressions that are involved in cell proliferation and migration [18].

In the present study, we compared the levels of AJ, TJ, and GJ proteins in CAOMECS graft; in healthy and normal rabbit corneal epithelial cells; and in corneal epithelial surface cells present after experimentally induced LSCD. The distribution of these junctional complexes was also investigated in rabbit corneas that received CAOMECS for treatment of experimentally induced LSCD. 

## 2. Materials and Methods

### 2.1. Animal Studies

New Zealand white rabbits weighing between 2.5 and 3 kg were used. They were maintained according to the Guidelines of Animal Care, as described by the National Academy of Sciences and published by the Institute of Laboratory Animal Resources Commission on Life Sciences National Research Council.

### 2.2. LSCD Model and CAOMECS Grafting

The experimental protocol was approved by the IACUC and performed as previously reported [13]. Briefly, the protocol was established with the following schedule: (1) anesthesia, (2) LSCD creation that was surgically accomplished by performing a 360-degree superficial lamellar dissection of the limbal zone, (3) follow-up and corneas exams were performed for up to three months, (4) oral mucosa biopsy, (5) epithelial cells isolation and primary cell culture, (6) CAOMECS grafting onto cornea surface that was accomplished using 3 to 5 sutures to secure the sheet onto cornea surface, (7) follow-up, (8) grading of corneal opacification and superficial visualization, and (9) sacrifice of the experimental animals 6 months after CAOMECS graft [13].

### 2.3. Corneal Epithelial Cell Sampling

Control and LSCD rabbits were lightly sedated and corneas were exposed to 20% isopropyl alcohol for one minute to remove corneal epithelium from underlying Bowman's membrane. Corneal epithelium was then rinsed with sterile saline. Scraping and removal of all visible corneal epithelial cells were performed using a Crescent knife 2 mm angled, double bevel (Katena Products Inc.; Denville, NJ). The cells were collected in PBS, centrifuged, and then resuspended and lysed in protein lysis buffer for subsequent biochemical analysis. The samples were then frozen at −80°C until all samples, healthy and LSCD's epithelial cells, were gathered for biochemical analysis.

### 2.4. Oral Mucosa Epithelial Cell Isolation and Cell Culture

A small oral mucosal biopsy was performed on a sedated animal, using a 6 mm diameter disposable punch biopsy instrument (Biopunch HealthLink, Jacksonville, FL). The biopsy specimen was washed in sterile saline, sanitized in povidone iodine, washed again in sterile saline, and then washed in DMEM cell culture media. The specimen was then used to isolate oral mucosal epithelial cells, as previously described [13]. Briefly, the specimen was incubated with Dispase I for one hour at 37°C (Roche Diagnostics GmbH, Mannheim, Germany); the epithelium was separated from the* lamina propria* and then subjected to trypsin digestion to separate the epithelial cells. The isolated primary epithelial cells were then seeded at 5 × 10^5^ density on UpCell®-insert, a temperature-responsive culture dish (CellSeed Inc., Tokyo, Japan), and cocultured with mitomycin C- (MMC-) treated NIH/3T3 feeder cells. After 4 days of cell culture, the media were changed and EGF was added at 10 ng/mL final concentration. Cell culture media were changed every two days. After 2 weeks of growth, CAOMECS were harvested by reducing the culture temperature to room temperature for 40 min. Two CAOMECS were produced from each biopsy. The first one was used for grafting onto LSCD corneas, as previously reported [13], and the second one was used for biochemical analysis.

### 2.5. Functional Barrier of CAOMECS

1. Calcium rigidifies the extracellular E-cadherin domain. In the absence of calcium, E-cadherin loses its affinity for the facing E-cadherin which causes cells to lose their adhesion to each other. EGTA was used to chelate calcium and destabilize E-cadherin anchoring bridges between cells. Fully grown CAOMECS were incubated after two weeks with EGTA from sigma at 2.5 mM and 5 mM dissolved in cell culture media for 24 hours. CAOMECS were then harvested and cell lysates were analyzed for E-cadherin levels.
2. Rabbit oral mucosa epithelial cell sheets were cocultured for two weeks in Transwell® insert permeable support (24 mm insert, 0.4 *μ*m; Corning Incorporated, Kennebunk, ME) with NIH-3T3 feeder cells at the bottom of the 6-multiplate well. When CAOMECS were fully grown, they were incubated with Cascade Blue at 20 *μ*M for 24 hours. CAOMECS were then harvested and fluorescence was measured in 1 *μ*g of total protein from cell media and from CAOMECS cell lysates. Controls were CAOMECS cultured and harvested without Cascade Blue addition. Fluorescence was measured using a Perkin Elmer LS 30 spectrofluorometer at *λ* excitation 370 nm and *λ* emission: 430 nm.

### 2.6. Impression Cytology

Millicell® culture inserts 12 mm diameter, 0.4 *μ*m pore size, with mixed cellulose membrane (Millipore, Billerica, MA) were used to sample corneal surface cells. The inserts were placed on the cornea for few seconds and then slowly removed. The inserts were then allowed to dry for 1 h at room temperature. The inserts were then fixed in 10% neutral buffered formalin overnight and conserved in 70% ethanol prior to immune-histochemical staining analysis.

### 2.7. Immunohistochemistry

Harvested CAOMECS were fixed in 10% neutral buffered formalin, processed and paraffin embedded, sectioned, and stained with H&E or used for immunofluorescent staining using E-cadherin, N-cadherin, beta-catenin from (BD Bioscience, San Jose, CA. Lot # are 65490, 25721, and 63115, resp.), ZO-1 and CK4 (Santa Cruz Biotech., Santa Cruz CA, Lot # are # K0413 and # G1514, resp.), K6 (Abcam Cambridge, MA. Lot # GR112707-1), Cnx43 (Abcam Cambridge, MA, Lot # GR1969-5), Ki-67 (Santa Cruz Biotech., Santa Cruz CA, Lot # E1403), Muc5AC (LifeSpan Biosciences, Inc. Seattle, WA, Lot # 60020), K13 (Santa Cruz Biotechnology, Santa Cruz, Lot # E1711), and deltaNp63 (Biocare Medical, Concord, CA Lot # 120815) antibodies. Normal, sham and grafted rabbit cornea specimens were fixed in 10% neutral buffered formalin. The processed tissues were sectioned, slides were stained with H&E, and immunofluorescent staining was performed using the same antibodies listed above. Alexa Fluor® 488 donkey anti-mouse/goat fluorophore conjugated secondary antibodies were used. Propidium iodide (Invitrogen, Eugene, OR) or 4′,6-diamidino-2-phenylindole (DAPI) (Thermo Scientific, Waltham, MA) was also used for staining nuclear DNA. A Nikon 400 fluorescent microscope was used to analyze the slides. Picture processing and analysis were performed using Adobe Photoshop CS5.

### 2.8. Western Blot

Protein concentration was measured using a Bio-Rad protein assay. Five micrograms of total proteins from harvested and lysed CAOMECS was used and compared to 5 micrograms of healthy, normal rabbit corneal epithelium harvested from the cornea surface with scraping. Five micrograms of protein scraped and removed from the corneal surface of a rabbit eye with experimentally induced LSCD was also processed for western blot analysis. Cell homogenates were separated by SDS-PAGE electrophoresis using 4–20% gradient polyacrylamide gels. Proteins were transferred to a PVDF membrane (Bio-Rad, Hercules, CA) for 1 h in 25 mM Tris-HCl (pH = 8.3), glycine 192 mM, and 20% methanol. Antibodies against Phospho-Akt Ser473 and GAPDH (Millipore, Temecula CA Lot # 2089910), phospho beta-catenin (Ser33-Ser37-Th41) (Cell Signaling Technology BD Bioscience, San Jose, CA Lot # 3), and occludin (Invitrogen, Life technology, Grand Island, NY, Lot # 1578827A) were used. Goat anti-mouse and sheep anti-goat antibodies (Bio-Rad, Hercules, CA) were used as secondary antibodies. Immunodetection was done using ECL plus (Amersham Bioscience Corp., Piscataway, NJ). Densitometric measurements of the bands were done using the GS-800 imaging densitometer (Bio-Rad, Hercules, CA).

### 2.9. Statistics Analysis

Data were obtained from at least three separate experiments. Bars represent mean values ± SEM. *p* values are determined by one-way ANOVA and Student-Newman Keuls for multiple group comparisons (Sigma-Stat Software, San Francisco, CA). Statistical significance was set at *p* = or < to 0.05. Bar graphs were shown as mean ± SEM, *n* = 3-4.

## 3. Results

To test our hypothesis that CAOMECS, cultured on a temperature-responsive cell culture surface, has the characteristics of functional and safe corneal epithelium, we measured and histologically analyzed the levels and distribution of epithelial markers (E-cadherin, N-cadherin, beta-catenin, phosphorylated beta-catenin, phosphorylated Akt, ZO-1, occludin, and Cnx43).

**Figure 1:**
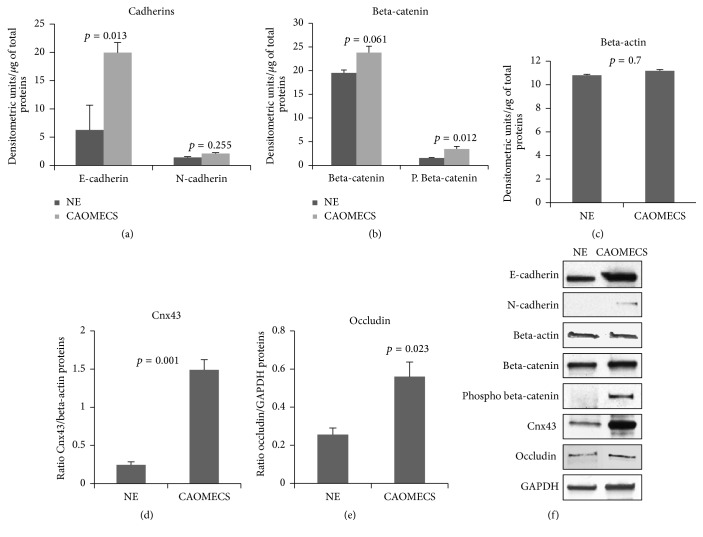
Western blot analysis of E-cadherin and N-cadherin (a), beta-catenin and phosphorylated beta-catenin (b), beta-actin levels (c), Cnx43 (d), and occludin (e) levels. (f) Pictures of targeted protein. CAOMECS: cultured autologous oral mucosa epithelial cell sheet; NE: rabbit healthy normal epithalial cells.

E-cadherin was significantly more expressed in CAOMECS graft compared to the corneal epithelium of a healthy and normal eye (NE). The level of expression of N-cadherin was much lower in both tissues (Figure 1(a)). Total beta-catenin levels measurements did not show a significant difference, while phosphorylated beta-catenin was increased in CAOMECS compared to NE, indicating greater turnover of beta-catenin in CAOMECS (Figure 1(b)). We then measured the levels of Cnx43 and occluding, both of which are important proteins in the junctional complexes of many cell types. Results showed that CAOMECS expressed high levels of these two junctional proteins (Figures 1(d) and 1(e)).

**Figure 2:**
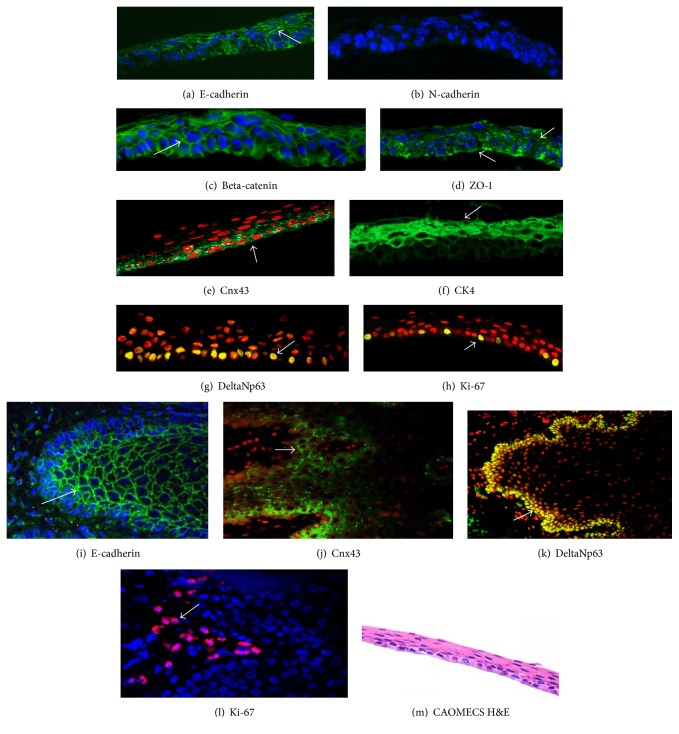
Immunofluorescent staining analyzing epithelial barrier and proliferation markers of CAOMECS (a to h) and control oral mucosal tissue sections (i to l). In green (magnification ×40) (a) and (i) are E-cadherin (arrow), (b) is N-cadherin, (c) is beta-catenin, and (d) is ZO-1. Nuclear staining is achieved using DAPI (blue) or propidium iodide (red). In green (magnification ×40), (e) and (j) are Cnx43 (arrow), (g) and (k) (magnification ×20) are DeltaNp63, and (h) and (l) are Ki-67 (arrow). (f) A staining for CK4 in green (magnification ×40). (m) is an H&E staining of CAOMECS.

The isolated oral mucosa epithelial cells (starting material) were not used as a control in the Western blot analysis because their junctional complexes were damaged by enzymatic treatment in the isolation procedures. Therefore, we used normal cornea epithelial cells instead and controls normal oral mucosa tissue sections were used in histological analysis. CAOMECS was histologically analyzed for E-cadherin, N-cadherin, beta-catenin, ZO-1, CK4, DeltaNp63, Cnx43, and Ki-67 (Figures 2(a)–2(h), arrows). Control oral mucosa sections stained positive for E-cadherin in squamous epithelial cells and stained negative in basal cells that stained positive for Cnx43, deltaNp63, and Ki-67 (Figures 2(i)–2(l), arrows). Histological analysis also showed that CAOMECS markedly stained for E-cadherin in the cell membrane, visualizing bridges that solidify adhesion between adjacent epithelial cells (Figure 2(a), arrow). CAOMECS did not stain positive for N-cadherin (Figure 2(b)). Our analysis also showed that beta-catenin, an E-cadherin downstream signaling protein, was located in the cytoplasmic membrane colocalizing with E-cadherin, which further documents the formation of AJ in CAOMECS (Figure 2(c)). TJ protein ZO-1 plays a major role in epithelial cell adhesion by connecting beta-catenin and intermediate filaments to gap junction proteins Cnx43 and 45 [19]. ZO-1 highlighted the intercellular bridges in CAOMECS (green and in a spotty staining, Figure 2(d) arrows). Cnx43 also stained CAOMECS intercellular space in green around the basal cells (Figure 2(e), arrow), almost similar to deltaNp63 and Ki-67 staining (Figures 2(g) and 2(h), arrows). CAOMECS epithelial differentiation was analyzed by Cytokeratin 4 (CK4) staining showing that only the apical squamous cells were positive for CK4 (Figure 2(f), arrow).  Figure 2(m) shows an H&E of a CAOMECS.

**Figure 3:**
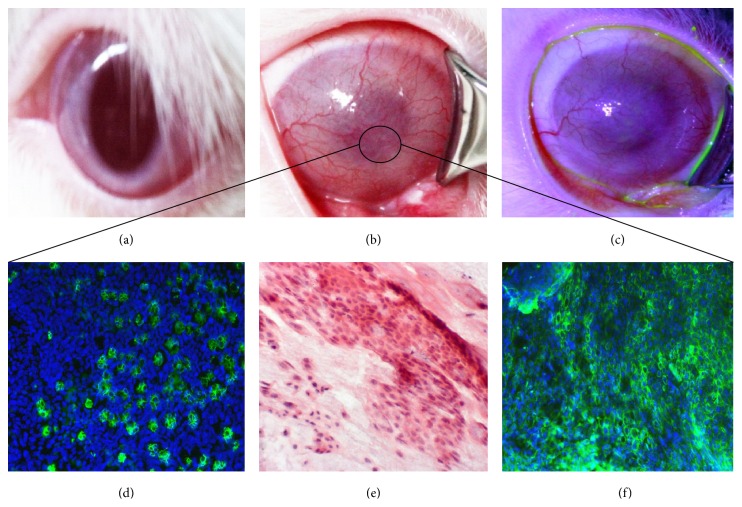
Rabbit LSCD model. (a) is a normal rabbit cornea. (b) and (c) are LSCD cornea 3 months after limbectomy, without and with fluorescein staining, respectively. (d) and (f) are corneal epithelium collected by impression cytology from the surface of the LSCD central corneal epithelium and subjected to immunofluorescent staining in green for Muc5AC and CK13, respectively. (e) is pannus tissue harvested from the LSCD cornea and processed for H&E staining. Blue is DAPI for nuclei staining. Note that LSCD cornea surface was invaded by conjunctival cells that stained positive for goblet cells (d) and for CK13 (f) indicating invasion of conjunctival epithelium over the cornea (magnification ×20).

An LSCD rabbit model was then created, as previously reported [13]. All superficial limbal tissue and other cornea epithelium were surgically removed. Follow-up exams were done monthly for 3 months after surgical limbectomy. Stabilized LSCD was evidenced by the neovascularization covering central cornea (Figure 3(b)), opaque corneal surface (conjuntivalization), and low presence of inflammatory cells, as indicated by impression cytology-based histological analysis (Figure 3(e)). Goblet cells were detected in central cornea with Muc5AC staining, as well as CK13 positive staining (Figures 3(d) and 3(f), resp.), reflecting the invasion of the naked stroma by the conjunctival epithelium, resulting in development of total LSCD.

**Figure 4:**
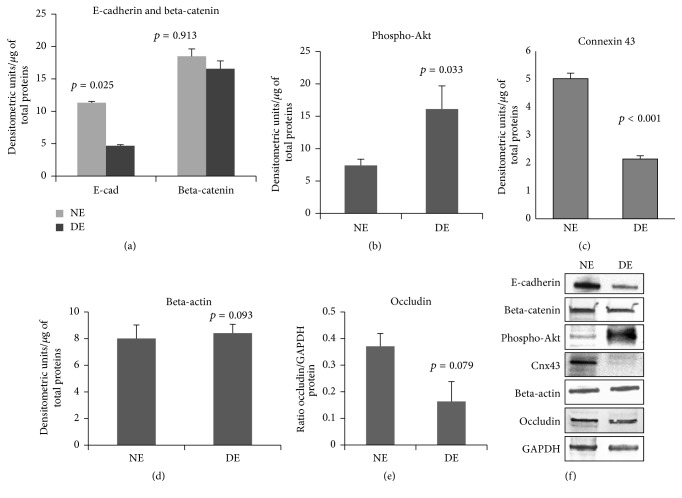
Western blot analysis of E-cadherin and beta-catenin (a); phospho-Akt (b), Cnx43 (c), beta-actin (d), and occludin (e) levels. Beta-actin and GAPDH (f) were used for loading control. NE: rabbit healthy epithalial cells. DE: LSCD's epithelium 3 months after limbectomy E-cadherin levels decreased significantly in the LSCD cornea while phosph-Akt levels were increased.

Once LSCD was stable 3 months after limbectomy, all of the corneal surface cells (including most pannus tissue) were removed with scraping and collected in lysis buffer for biochemical analysis. Results showed a significantly lower expression of E-cadherin in LSCD's epithelium and no significant changes in beta-catenin (Figure 4(a)). However, Akt was more phosphorylated in the LSCD's epithelial cells, suggesting a decrease in epithelial adhesion (Figure 4(b)). Upregulated E-cadherin exerts a negative control on phosphoinositide 3-kinase (PI3K)/Akt signaling activation by inhibiting beta-catenin downstream signaling, thus preventing epithelial cell decreased adhesion. The levels of Cnx43 were not found to be significantly different in normal rabbit corneal epithelium and LSCD's epithelia (Figure 4(c)). However, occludin levels were significantly decreased in LSCD cornea, compared to normal epithelium cornea (Figure 4(e)). The targeted protein bands are shown in Figure 4(f).

**Figure 5:**
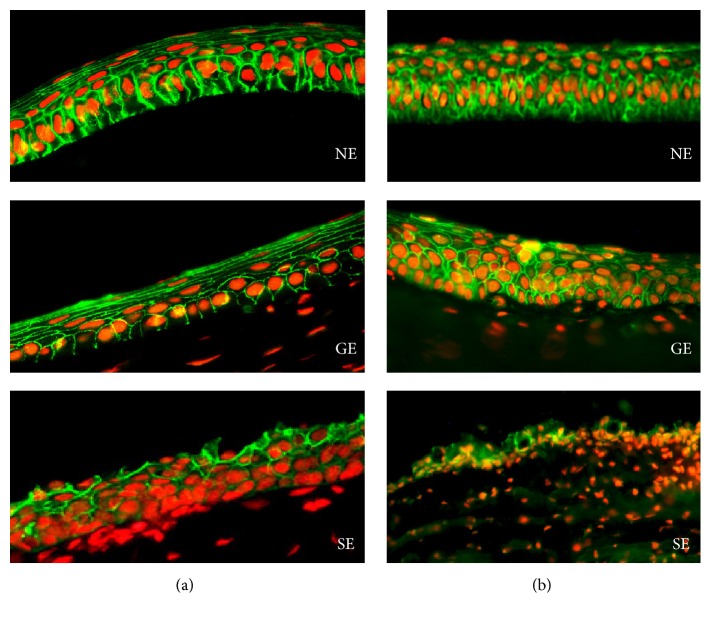
E-cadherin (a) and beta-catenin (b) expression is shown in green. Red is nuclear staining with propidium iodide. NE: normal cornea, GE: CAOMECS grafted cornea, and SE: sham cornea. Note that E-cadherin and beta-catenin stained markedly on the cell membrane of normal cornea and CAOMECS grafted corneas compared to that of the sham cornea (magnification ×40).

Three months after limbectomy, rabbit's oral mucosa was biopsied and used to engineer CAOMECS graft. The CAOMECS graft was then used for grafting onto a rabbit cornea with LSCD. After 6 months of follow-up and eye exams, all corneas were analyzed for junctional complexes. In our recently published study [13], we reported the success of CAOMECS grafting and showed reepithelialization of corneas with no epithelial defect. Grafted corneas showed an E-cadherin expression pattern (Figure 5(a)) similar to that of normal healthy corneas (Figure 5(a)). However, sham-operated corneas showed a disrupted E-cadherin staining and a decrease of its expression mainly at the basal cell membrane (Figure 5(a)), indicating an unhealthy epithelium. CAOMECS grafted corneas (Figure 5(b)) also showed beta-catenin staining concentrated at the cell membrane, similar to healthy corneas (Figure 5(b)), while the sham-operated cornea did not show well-defined beta-catenin cell membrane staining (Figure 5(b)). This result was expected as the sham eyes showed cornea epithelial thinning and erosion, as well as significant goblet cell invasion (Figures 5(a) and 5(b)).

**Figure 6:**
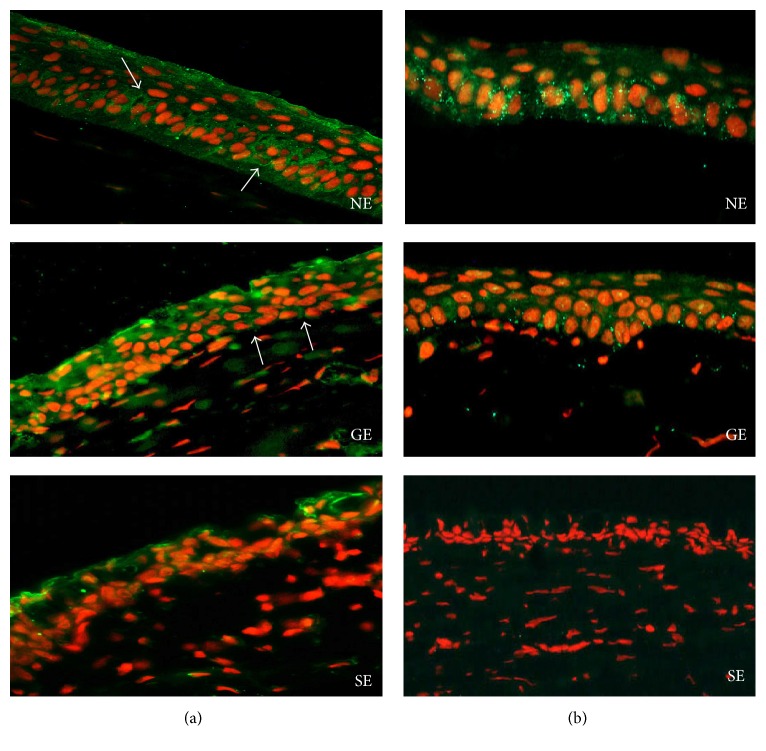
ZO-1 (a) and Cnx43 (b) expression is shown in green. Red is nuclear staining with propidium iodide. NE: normal cornea, GE: CAOMECS grafted cornea, and SE: sham cornea (magnification ×40).

The corneal epithelium is the main barrier protecting the eye from harmful external agents, such as microbes and chemicals, and contributes to the transparency of the cornea. This barrier function depends on TJs that seal the intercellular space. TJ molecule ZO-1 stained positive between adjacent CAOMECS epithelial cells (Figures 2(d) and 2(e)). CAOMECS grafted cornea also showed a positive staining of ZO-1 at the cell junctions (Figure 6(a)), while the sham eye tissue section showed an uneven and discontinuous ZO-1 staining (Figure 6(a)). As expected, these interactions were stabilized by intercellular tethering forces generated by the AJs, which are proximal to TJs, thus reflecting a normal functional epithelial barrier.

**Figure 7:**
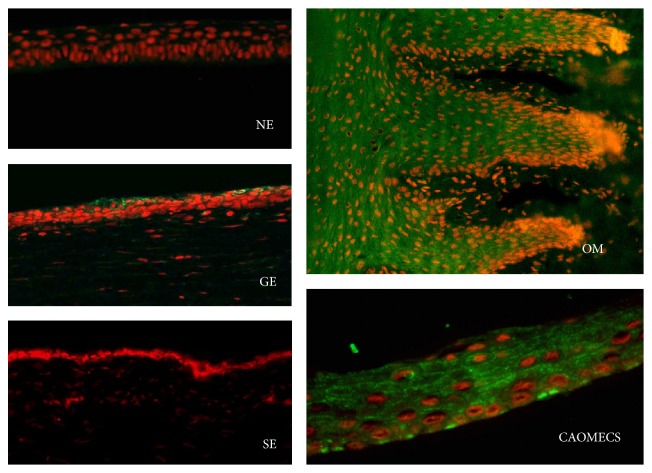
Cytokeratin 6 (K6) staining is shown in green. Red is nuclear staining with propidium iodide. NE: normal cornea, GE: CAOMECS grafted cornea, and SE: sham cornea. OM is oral mucosa (corneas and OM tissue sections magnification is ×20 and CAOMECS magnification is ×40).

Cnx43 was mainly found in the basal cell layer of normal corneal epithelium, as well as CAOMECS grafted corneas (Figure 6(b)), while the sham corneas did not show any Cnx43 staining (Figure 6(b)). These results reflected the beneficial effects of CAOMECS grafting onto corneas of rabbit with LSCD. Evidence of oral epithelial cells existence in CAOMECS grafted corneas six months after the grafting was also investigated.  Figure 7 documents the existence of these cells using Cytokeratin 6 (K6) biomarker expressed in oral mucosal epithelial cells, but not on the corneal epithelium. CAOMECS grafted corneas showed scattered or spotted positive staining of K6, indicating the presence of oral mucosal epithelial cells on the ocular surface even 6 months after grafting (Figure 7).

**Figure 8:**
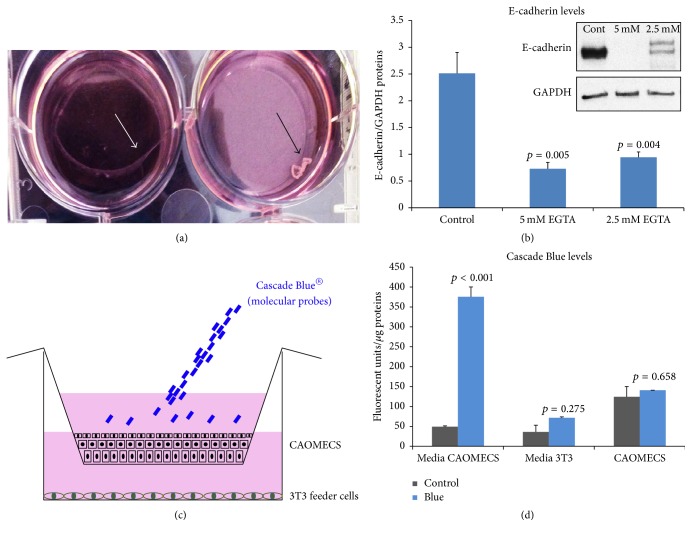
E-cadherin role in CAOMECS epithelial barrier. (a) Picture of CAOMECS commencing to detach when treated with 2.5 mM EGTA (white arrow) and completely lost adhesion when treated with 5 mM EGTA (black arrow). (b) is a semiquantitative measurement of E-cadherin. Note that E-cadherin levels were significantly reduced when CAOMECS grafts were treated with EGTA. (c) is an illustration of Cascade Blue experiment design where the probe was only added in cell culture media of CAOMECS. The graph in (d) showed the levels of Cascade Blue in media and in CAOMECS cells compared to controls (CAOMECS culture without Cascade Blue addition). Note that fluorescence did not traverse the barrier of CAOMECS.

Calcium covalently binds to Ca^2+^ binding domain, present in the N-Terminal of E-cadherin, allowing bridging between neighboring cells. When calcium is removed, E-cadherin molecule becomes disordered and easily denatured, causing E-cadherin molecules to dissociate. EGTA was used to chelate extracellular calcium and demonstrate E-cadherin role in epithelial integrity and thus epithelial barrier. CAOMECS were cultured for two weeks and treated with EGTA for 24 hours before harvest. CAOMECS commenced to detach in 2.5 mM EGTA treatment (Figure 8(a) white arrow) and was completely detached in 5 mM EGTA treatment (Figure 8(a) black arrow). A semiquantitative analysis of E-cadherin levels showed a significant decrease of E-cadherin after Ca^2+^ chelation by EGTA (Figure 8(b)). In addition, the epithelial barrier function of CAOMECS graft was investigated using a small fluorescent probe, Cascade Blue hydrazide that does not penetrate cells unless there is a destabilization of cell membrane (Figure 8(c)). Results showed a high concentration of fluorescence in CAOMECS culture media and no fluorescence in the cells. Moreover, there was no fluorescence in the culture media of the 3T3 feeder cells or in the 3T3 cells by themselves, indicating that CAOMECS exerted a barrier to Cascade Blue molecule (Figure 8(d)).

## 4. Discussion

CAOMECS is a successful technology for ocular surface reconstruction in cases of preexisting limbal stem cell deficiency (LSCD); its efficacy has been demonstrated by several groups of investigators [1, 20]. Since E-cadherin signaling is important for establishing all cell-to-cell junctional complexes, the present study investigated E-cadherin signaling in CAOMECS before and after grafting on corneas of rabbit with experimentally induced LSCD. We examined junctional complexes and compared the expression levels of these proteins between CAOMECS, normal corneas, LSCD corneas, and CAOMECS grafted corneas.

E-cadherin is an AJ calcium-dependent protein that plays an important role in cell adhesion, binding cells together and regulating intracellular downstream signaling. These bindings increase cytoskeleton interaction with tight and gap junction proteins, thus increasing cell adhesion. Abundant literature shows that E-cadherin is involved in cell polarity [21–23], cell differentiation [24, 25], and cell proliferation [26]. In the present study, we found that E-cadherin was significantly expressed in CAOMECS, at higher levels than normal healthy epithelial cells. This upregulation was consistently reproducible among all the CAOMECS grafts we engineered and suggests that there are relatively strong adhesions between cells in CAOMECS grafts. Note that cell culture conditions and more specifically cell culture media containing calcium are responsible for E-cadherin stabilization at the cell membrane. Indeed, when calcium was chelated with EGTA, CAOMECS completely lost adhesion to cell culture surface, and the levels of E-cadherin were significantly reduced. This finding indicated that calcium supplementation in the cell culture media promoted E-cadherin expression and bridging neighboring cells, which almost certainly increases the epithelial barrier function of CAOMECS graft.

E-cadherin expression is also regulated at the transcriptional level and by cytoplasmic proteasome degradation [27–29]. Our previous study showed that CAOMECS proteasome activity levels were higher than those of healthy cornea epithelial cells [30], leading to E-cadherin stabilization at the cell membrane [31].

E-cadherin/beta-catenin complex plays an important role in cell adhesion and in the formation of the cell skeleton. Loss of E-cadherin/beta-catenin complex from the cell membrane is commonly observed in hyperproliferative epithelial cells [29]. Wnt pathway acts through the beta-catenin signaling pathway to promote angiogenesis and tumorigenesis if the pathway is unregulated [32, 33]. This occurs through beta-catenin translocation to the nucleus and activation of T-cell factor/lymphoid enhancer factor (TCF/LEF) transcription factors gene expression, consequently increasing cell proliferation [34]. When E-cadherin intracellular downstream signaling was analyzed by comparing the levels and distribution of beta-catenin, results showed that CAOMECS had similar levels to those of healthy corneal epithelial cells. Moreover, we found phosphorylated beta-catenin levels higher in CAOMECS when compared to healthy cornea epithelial cells, indicating that beta-catenin was targeted for proteasome degradation [35]. Beta-catenin phosphorylation leads to its ubiquitination, followed by degradation by the proteasome, thus preventing nuclear translocation and TCF genes activation for cell prosurvival behavior [36]. An important function of membranous beta-catenin is to recruit alpha-catenin, which, in turn, recruits cytoskeleton filaments as well as TJ proteins ZO-1 and occludin. The AJ proteins, together with TJ's, form the apical junction complex that controls epithelial cell-to-cell adherence, barrier function, and regulation of the actin cytoskeleton as well. TJs are the most apical junction complex and provide a functional barrier [37]. They are composed of transmembrane proteins occludin and claudin, which anchor scaffolding proteins, including ZO-1, ZO-2, and ZO-3. Both AJs and TJs have intricate connections and play a major role in maintaining the integrity of the epithelial barrier [38]. The staining of ZO-1 showed results similar to the results of E-cadherin staining at the cell membrane. We showed positive staining of ZO-1 at the plasma membrane of CAOMECS grafted corneas, indicating that CAOMECS grafting reepithelized the cornea surface with cells containing expressed tight junction proteins. Moreover, occludin was expressed in CAOMECS, indicating that TJ proteins were formed in CAOMECS in a quantity and pattern of distribution similar to normal cornea epithelial cells. By binding to occludin, ZO-1 can bind actin filaments and could possibly serve as a cadherin-associated protein to link nascent adhesions to the actin cytoskeleton [39, 40].

Our results showed high levels of E-cadherin at the plasma membrane of CAOMECS and subsequent downstream signaling of recruited beta-catenin, actin filaments, TJ proteins, and gap junction proteins. These results suggest that CAOMECS grafting allowed the corneas to be reepithelialized with a sheet of multilayered cells that may have a barrier function similar to other types of normal epithelial tissue. However, the literature reports various results for oral mucosal epithelial cells functional barrier [4, 7–9]. It is possible that the novel cell culture technique we used resulted in a different outcome than these reports. It is also possible that oral mucosal epithelial cells are permeable to fluorescein sodium. However, fluorescein has a relatively small molecular weight of 332.306 Daltons. Testing the epithelial barrier function with a low molecular weight molecule does not always give the same result as when the barrier function is tested with larger molecular weight molecules [4]. TJ's paracellular diffusion barrier is semipermeable as it allows the passage of small size hydrophilic molecule and ions [41]. Other investigators have demonstrated that oral mucosal epithelial cell sheets grown in a cell culture plate with a temperature-responsive surface temperature-responsive surface have a functional barrier [7, 9, 13]. Our present study showed that CAOMECS graft had significantly higher levels of TJ proteins when compared to control corneal epithelium. These results suggest that CAOMECS graft may have the properties to prevent epithelial defects and ulceration and create a functional barrier that accelerates and improves corneal surface healing [13]. Similar to skin, oral mucosal epithelium is an example of the toughest and most protective epithelium. In this study, we also used Cascade Blue hydrazide from Molecular Probes in our mechanistic approach to confirm the barrier function of CAOMECS graft. This fluorescent dye is a water soluble fluorescent probe with low molecular weight (<1000 Daltons) and is too polar to passively diffuse through cell membranes. The barrier that CAOMECS graft exerted on Cascade Blue passage through the multilayered CAOMECS grafts and the barrier against Cascade Blue diffusion into CAOMECS cells confirm the barrier function of CAOMECS. In our previous study [13], we showed that CAOMECS reduced the number of blood vessels and prevented the regrow of pannus tissue in the central cornea of rabbits with experimentally induced LSCD. However, whether CAOMECS constitutes a barrier to external toxic and infectious agents is not known and remains to be determined.

It is well known that the E-cadherin/beta-catenin complex regulates gap junction proteins, such as Cnx43, because E-cadherin/beta-catenin complex loss was found associated with Cnx43 loss at the cell membranes [42, 43]. Cnx43 levels and expression in CAOMECS grafts were similar to normal corneal epithelium. Histological analysis of CAOMECS also demonstrated the location of these gap junction proteins at their appropriate intercellular sites and showed the expected expressions of epithelial marker. Corneal epithelial cells from rabbits with LSCD showed low expression in Cnx43, E-cadherin, and beta-catenin levels, reflecting a disruption of cell adhesion, probably caused by inflammation, wound healing, and fibrosis. In addition, phosphorylated Akt was significantly upregulated, indicating phosphoinositide 3-kinase (PI3K)/Akt signaling activation for cell migration [44, 45]. Because of the potential for cell migration and to further document CAOMECS safety, we plan to track possible migration of individual cells in CAOMECS with a direct labelling approach [46].

CAOMECS grafted corneas showed the staining of E-cadherin and beta-catenin at the cell membrane, while sham corneas showed a weaker staining of this complex. Our morphological analysis also showed a positive staining of Cnx43 in CAOMECS grafted corneas, similar to that of normal healthy corneas. The sham corneas (with induced LSCD but no CAOMECS graft) did not stain positive for Cnx43, suggesting relatively weak cell-to-cell adhesion and a lack of cell-cell communication [43]. In healthy corneal epithelium, Cnx43 was mainly found in the limbus and cornea epithelial basal cells, in a pattern very similar to deltaNp63 staining, which suggests there might be a role for Cnx43 in progenitor stem cells [47, 48]. We also found Cnx43 in CAOMECS grafted cornea basal cells. In conclusion, we used E-cadherin expression as an indicator of a functional epithelial barrier because E-cadherin facilitates intercellular adhesion and recruits catenin proteins and actin filaments to cell borders to increase barrier function. CAOMECS graft engineered on temperature-responsive surface allowed the production of multilayered epithelial cell sheets with the expression of epithelial markers necessary for normal corneal epithelial barrier function.

## Abbreviations

CAOMECS: Cultured autologous oral mucosa epithelial cell sheet
LSCD: Limbal stem cell deficiency
Zo-1: Zonula occludens protein 1
Cnx43: Connexin 43
EGTA: Ethylene glycol-bis (*β*-aminoethyl ether)-N,N,N′,N′-tetraacetic acid.
